# Choking and the Advantages of Belladonna in Treatment of Same

**Published:** 1894-02

**Authors:** John Meikle

**Affiliations:** Larne


### CHOKING AND THE ADVANTAGES OF BELLADONNA IN TREATMENT OF SAME.

By John Meikle, M.R.C.V.S., Larne.

The subject of above was a cow the property of a dairyman of this town, the breed of which it would be almost an impossibility to describe, notwithstanding the protests of the owner as to her having the best of pedigrees—they have all good qualities and pedigrees, I may remark, when critically unwell.

This cow had been in good health up to the 29th December, when she was allowed the privilege of a run in the yard, convenient to the byre. It happened unfortunately that she came across a basket of raw potatoes to which she began helping herself too lavishly, with the result that in bolting the delicacies she contrived to settle one in the cervical region of the œsophagus. For five hours the owner had endeavored to remove the obstruction without success, and eventually I was sent for, about 6 P. M., to relieve the suffering animal, if possible. On my arrival the cow was down; she appeared to be pretty well exhausted, and in great agony, with an anxious expression of countenance. Water and other fluids were returned through the nose and mouth, and with accompanying periodical attacks of coughing. Pulse 120, weak, irregular, temperature 102 F., with increased respirations, and sweats over the body. The rumen had been punctured without giving the desired relief. The foreign body could be distinctly felt giving rise to an enlargement on the left side, in the region of the œsophagus, around which the tissues and the circular muscular fibres above and below the obstruction were swollen, rigid and unyielding. Without doubt the symptoms were aggravated by unnecessarily rough treatment.

I could see after examining my patient that treatment must be prompt to prove successful, so got the cow into position, passed the probang by ordinary method, taking all the necessary precautions, but found I could not encourage the obstruction to move either by pressure, or gentle tapping combined with external manipulation. Keeping the probang in position, I dissolved three drachms of extract of belladonna in glycerine, and compounded a suitable emulsion with warm water ; and down the probang I poured the preparation, after removing the stiletto. At the same time I injected with difficulty some of the preparation with a hypodermic syringe on the side of and around the potato, so that the drug came into direct contact with the tissues around the exciting cause, and after applying hot cloths to the outside of the swelling I had the satisfaction of seeing the obstruction move on. The cow jumped to her feet within fifteen minutes and partook of some linseed tea. I met the owner a few days afterwards, and happening to enquire about the cow, I elicited the remark that she was "doing rightly."

I am of opinion that the belladonna and the hot water cloths caused the muscular fibres of the œsophagus to relax, and thus favor the passage of the potato.—*The Veterinary Record.*

